# TET-mediated DNA hydroxymethylation is negatively influenced by the PARP-dependent PARylation

**DOI:** 10.1186/s13072-022-00445-8

**Published:** 2022-04-05

**Authors:** Anja Tolić, Mirunalini Ravichandran, Jovana Rajić, Marija Đorđević, Miloš Đorđević, Svetlana Dinić, Nevena Grdović, Jelena Arambašić Jovanović, Mirjana Mihailović, Nataša Nestorović, Tomasz P. Jurkowski, Aleksandra S. Uskoković, Melita S. Vidaković

**Affiliations:** 1 Department of Molecular Biology, Institute for Biological Research “Siniša Stanković”, National Institute of Republic of Serbia, University of Belgrade, Bulevar despota Stefana 142, 11060 Belgrade, Serbia; 2 Institute of Biochemistry, University of Stuttgart, Pfaffenwaldring 55, 70569 Stuttgart, Germany; 3 Department of Anatomy, University of California, San Francisco, 513 Parnassus Avenue, HSW 1301, San Francisco, CA 94143 USA; 4 Department of Cytology, Institute for Biological Research “Siniša Stanković”, National Institute of Republic of Serbia, University of Belgrade, Bulevar despota Stefana 142, 11060 Belgrade, Serbia; 5 School of Biosciences, Cardiff University, Cardiff, Wales, Sir Martin Evans Building, Museum Avenue, Cardiff, CF10 3AX UK

**Keywords:** DNA demethylation, 5-Hydroxymethylcytosine, PARP, TET, PARylation

## Abstract

**Background:**

Poly(ADP-ribosyl)ation (PARylation), a posttranslational modification introduced by PARP-1 and PARP-2, has first been implicated in DNA demethylation due to its role in base excision repair. Recent evidence indicates a direct influence of PARP-dependent PARylation on TET enzymes which catalyse hydroxymethylation of DNA—the first step in DNA demethylation. However, the exact nature of influence that PARylation exerts on TET activity is still ambiguous. In our recent study, we have observed a negative influence of PARP-1 on local TET-mediated DNA demethylation of a single gene and in this study, we further explore PARP–TET interplay.

**Results:**

Expanding on our previous work, we show that both TET1 and TET2 can be in vitro PARylated by PARP-1 and PARP-2 enzymes and that TET1 PARylation negatively affects the TET1 catalytic activity in vitro. Furthermore, we show that PARylation inhibits TET-mediated DNA demethylation at the global genome level in cellulo.

**Conclusions:**

According to our findings, PARP inhibition can positively influence TET activity and therefore affect global levels of DNA methylation and hydroxymethylation. This gives a strong rationale for future examination of PARP inhibitors' potential use in the therapy of cancers characterised by loss of 5-hydroxymethylcytosine.

**Supplementary Information:**

The online version contains supplementary material available at 10.1186/s13072-022-00445-8.

## Background

DNA damage poses a constant threat to genome integrity. In cells, different types of DNA damage are repaired by enzymatic machinery belonging to several well-coordinated repair pathways [1, 2]. The earliest event in DNA damage repair is the signalisation of diverse types of DNA lesions managed by the recruitment of poly(ADP-ribose) polymerase-1 (PARP-1). PARP-1 is the most prominent member of the PARP family of enzymes, and together with PARP-2, can introduce a covalent posttranslational modification on target proteins (including the PARP proteins themselves) by forming linear or branched poly(ADP-ribose) (PAR) polymers on their surface, in a process known as poly(ADP-ribosyl)ation (PARylation). In addition, the flexibility and size of PAR polymers provide a significant enhancement in non-covalent binding to specialised PAR-binding motifs of various target proteins. Such multiprotein complexes, formed through non-covalent binding to PAR polymers, have functional consequences driven by PAR-responsive cellular pathways [3]. Furthermore, PARylation has been implicated in DNA demethylation due to its role in base excision repair (BER) [4, 5], but it can also be involved in DNA demethylation independently of its role in the DNA damage repair [6].

The interchange between DNA methylation and demethylation as essential epigenetic modifications is also essential for genomic integrity maintenance. DNA methylation patterns (5-methylcytosine (5mC)) are established and maintained by DNA methyltransferases (DNMTs) while DNA demethylation is initiated by the ten-eleven translocation (TET) proteins. The TET enzymes (TET1, TET2, and TET3) belong to a large family of Fe^2+^/α-ketoglutarate-dependent oxygenases that can initiate DNA demethylation by catalysing the oxidation of 5mC to 5-hydroxymethylcytosine (5hmC) [7, 8] and further oxidised bases 5-formylcytosine (5fC) and 5-carboxylcytosine (5caC). Besides being an intermediate in DNA demethylation, 5hmC is also considered to have regulatory functions as an independent epigenetic mark [9]. Kafer and co-workers [10] recently published that 5hmC localises to sites of DNA damage and repair and colocalises with major DNA damage response proteins (53BP1 and gH2AX), revealing 5hmC as an epigenetic marker of DNA damage. The fact that active DNA demethylation may occur specifically at sites of DNA damage is further supported by others [11, 12], who also suggested that TET enzymes and/or 5hmC may have a direct role in DNA repair.

Emerging evidence suggests that PARP-1 participates in the regulation of the levels of 5mC modifications [6, 13, 14], whilst TETs are essential for the oxidation of 5mC and thereby for the initiation of the DNA demethylation. This suggests that PARP and TETs family of enzymes work in concert to regulate DNA (de)methylation state and probably suggests their involvement in DNA damage repair. PARylation participates in transcriptional and posttranscriptional regulation of TET1, stimulating TET1 gene expression through epigenetic regulation and ensuring TET1 protein stability [15, 16]. Also, TET1 can be PARylated [17] or recruited to specific sites in the genome through non-covalent interaction with PAR polymers [18]. PARP-1 was found to interact with all members of the TET family [19] as well as potentially affect TET-mediated demethylation and hydroxymethylation of DNA [6, 17]. The direct impact of PARylation on TET1 hydroxylase activity was investigated and opposite effects were detected, depending on the nature of PAR polymer binding. Namely, covalent modification—PARylation, stimulated TET1 activity, while non-covalent interaction with PAR polymers led to its inhibition [17].

Our previous work indicated that PARP-1 affects TET activity on the local level of a single gene coding for the CXCL12 chemokine [20, 21], propelling us to further explore PARP/TET cross-talk on a global level by specifically looking into the influence that PARP activity exerts on TET-mediated DNA hydroxymethylation/demethylation. It has been proposed that excessive TET activity can induce DNA damage as active DNA demethylation involves forming abasic sites in the last steps of the process [22, 23]. Therefore, we hypothesised that PARPs, being involved in DNA repair through damage sensing, may negatively influence TET activity to prevent the potential accumulation of harmful levels of DNA breaks. In line with our hypothesis, we found that PARP-1-dependent PARylation inhibits the catalytic activity of TET1 in vitro, which was confirmed by the observed decrease of global DNA methylation level and concomitant increase of DNA hydroxymethylation upon inhibited PARylation in cellulo. These results showed a direct link between PARP activity and TET-mediated hydroxymethylation, proving that PARP-dependent PARylation can inhibit TET-dependent DNA hydroxymethylation and demethylation genome-wide. In addition, the results of our study also have translational potential as they introduce the possibility of future use of PARP inhibitors as therapeutic agents or adjuvants in a significant subset of cancers where the activity of TETs is disturbed towards decreased hydroxymethylation.

## Results

### In vitro PARylation of TET1 and TET2 proteins by PARP-1 and PARP-2

**Fig. 1:**
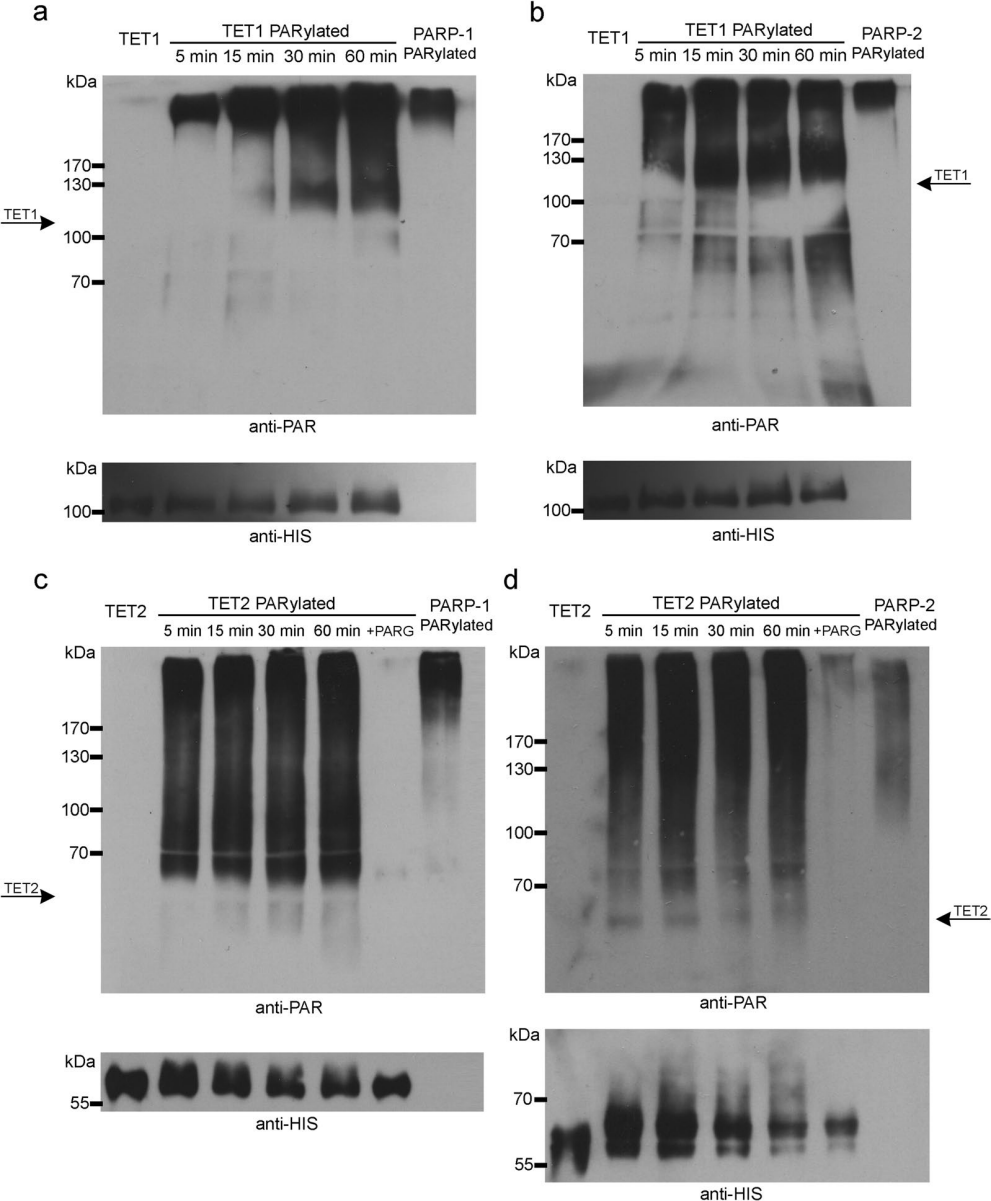
Kinetics of in vitro PARylation of TETs. Immunoblot detection of in vitro PARylation of TET1 by **a** PARP-1 and **b** PARP-2 and of TET2 by **c** PARP-1 and **d** PARP-2, with anti-PAR or anti-HIS antibody. Control samples—unmodified TET1 or TET2, in vitro auto-PARylated PARP-1 or PARP-2 and PARG treated PARylated TET2

To test whether murine mTET1 and mTET2 are PARylated by the PARP enzymes, we incubated in vitro the recombinant murine mTET1 and mTET2 catalytic domains with the recombinant human PARP-1 or PARP-2 in the presence of co-substrate NAD^+^. We have observed a time-dependent increase in PARylation signal above the position of the mTET1 and mTET2 catalytic domains, as determined by Ponceau staining. PAR polymers are covalently attached to the target proteins, and besides adding additional mass, they also add a negative charge that slows down the modified protein movement during electrophoresis resulting in an upwards stretching signal as observed. PARylation of both mTETs was detected by the presence of PAR signal extending from the mTET1 and mTET2 proteins’ positions towards the top of the film (Fig. 1 and Additional file 1: Fig. S1). We note that the signal obtained with the anti-HIS antibody is weaker than that detected with the anti-PAR antibody, potentially due to the smaller number of binding sites for the anti-HIS antibody and the smaller number of TET protein molecules (that have attached the longest and most branched PAR polymers) shifted to the highest positions.

Auto-PARylation of PARP-1 or PARP-2 is well described [24, 25] and part of the PAR signal originates from automodified PARPs indicating that the PARP proteins used are active. Based on a comparison with the control reaction in which only PARP is PARylated (for 60 min), all signal located between the positions of unmodified mTET and automodified PARP belongs to the in vitro PARylated mTET proteins (Fig. 1). Moreover, with the progression of the in vitro PARylation, the signal of PARylated mTET1 increased (Fig. 1a, b). For mTET2, a significant level of in vitro PARylation was already observed 5 min into the reaction with either PARP-1 or PARP-2 (Fig. 1c, d) showing greater PARylation efficiency compared to TET1. To further confirm the specificity of the PAR signal, we treated the previously PARylated mTET2 protein with a PARG enzyme that removes PAR polymers. Consequently, a noticeable loss of signal due to PAR polymers degradation could be observed, confirming successful mTET2 PARylation.

**Fig. 2:**
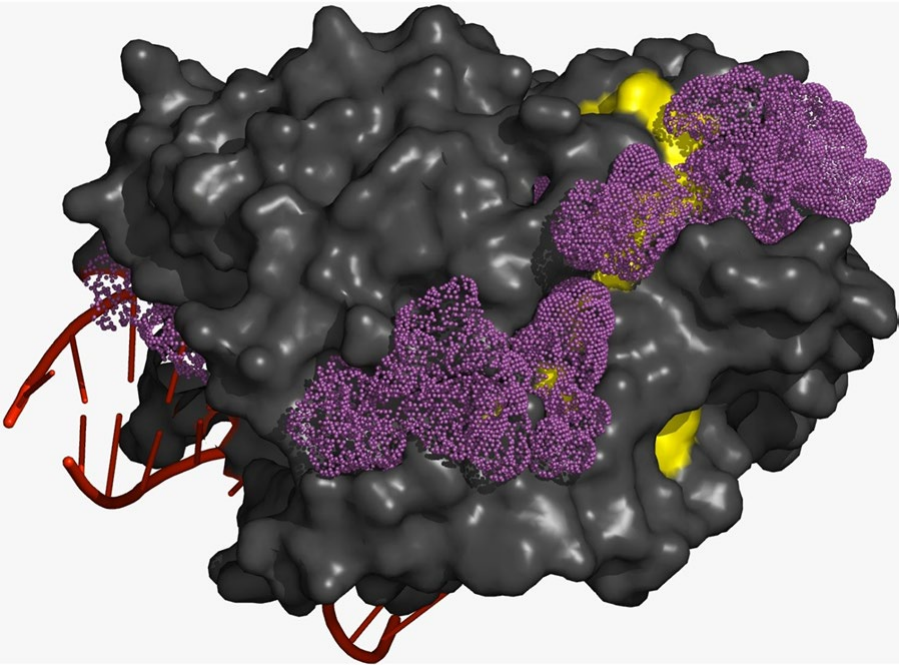
Docking model showing the crystal structure of human TET2 with predicted ATP binding sites. ATP docking (SwissDock) was performed to predict potential adenine binding sites on the surface of the protein. Grey—surface representation of TET2 protein (the DNA binding groove is on the opposite side of the protein); purple dots represent predicted top-scoring ATP binding sites; yellow colour denotes sequence motif observed in PAR-binding proteins [26]

Besides the covalent attachment of PARs, we assumed that TETs can be also non-covalently modified where PAR polymers are acting as mediators of protein–protein interactions. Using computational modelling (SwissDock), we mapped possible adenine binding pockets on the surface of the TET2 protein. Molecular docking suggested numerous possible binding sites on the protein’s surface. Significantly, an extended array of consecutive adenine binding sites can be observed on the protein surface side opposite to the DNA binding groove (Fig. 2). The observed distances between the neighbouring predicted adenine binding sites correspond perfectly to the theoretical distances between PAR units, suggesting that this groove is best suited to interact with a PAR polymer. In addition, a putative PAR-binding motif, composed of interspersed basic and hydrophobic amino acid residues (previously described by Pleschke et al. [26]), maps along this groove, providing further support for our assumption (Fig. 2).

### PARP-1-dependent PARylation inhibits TET1 activity in vitro

**Fig. 3:**
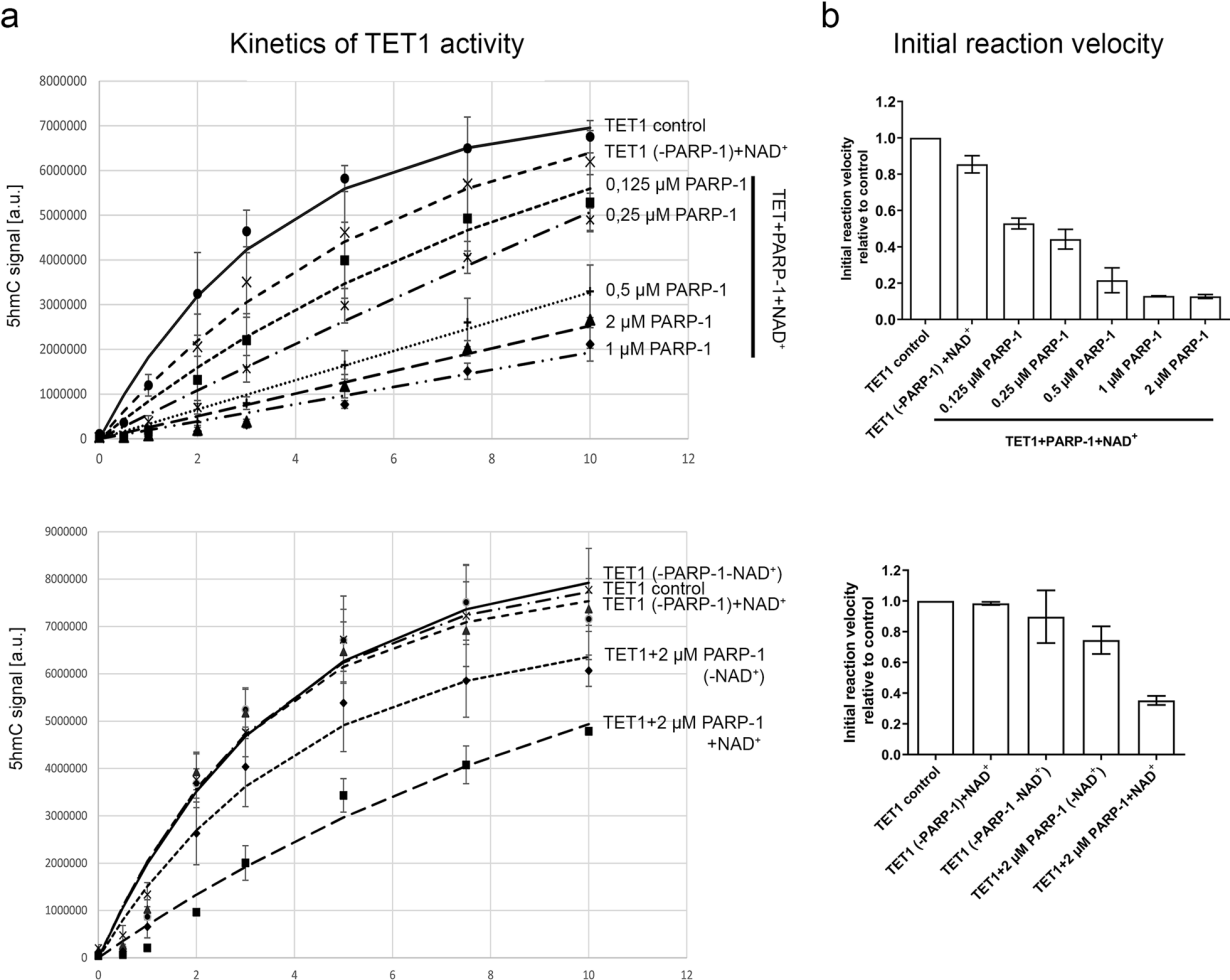
Influence of PARylatyion on TET1 activity in vitro**. a** Kinetics of TET activity of: in vitro PARylated TET1 (upper graph) and TET1 in control reactions (bottom graph). The level of 5hmC produced by TET1 was plotted as a function of time and exponentially fitted using the least square fit. The data points are shown as mean ± SEM (n = 2). **b** TET1 activity represented by initial reaction velocity of 5mC oxidation by: in vitro PARylated TET1 (upper graph) and TET1 in control reactions (bottom graph). Initial reaction velocities were calculated by linear regression based on the signal at the initial time points of TET activity kinetics. Results were scaled to TET1 control and shown as mean ± SEM (n = 2). TET1 control—unmodified TET1; TET1(-PARP-1) ± NAD^+^—TET1 mock PARylated with dialysis buffer instead of PARP-1 with or without NAD^+^; TET1 + PARP-1(-NAD^+^)—TET1 mock PARylated with PARP-1 without NAD^+^; TET1 + PARP-1 + NAD^+^—PARylated TET1. n‐number of independent experiments, a.u.-arbitrary units

Once we confirmed that the TET1 enzyme gets PARylated, we studied which effect this modification exerts on the TET1 enzymes' catalytic activity. First, we preincubated mTET1 with an increasing amount of PARP-1 protein and tested their 5mC oxidation activity with an ELISA-based assay [27] (Fig. 3). Significantly, it was observed that PARylation with increasing concentrations of PARP-1 progressively led to a decrease in mTET1 activity evidenced by reaction kinetics (Fig. 3a) and reduced initial reaction rate (Fig. 3b). On average mTET1 activity was decreased by 47% in reactions with 0.125 µM PARP-1 and an average of 87% decrease was detected in reactions with 1 and 2 µM PARP-1. A considerable decrease in the initial reaction rate of PARylated mTET1 was also observed compared to all control reactions (unmodified TET1, TET1 that underwent PARylation procedure with dialysis buffer instead of PARP-1 with/without PARP’s cofactor NAD^+^, TET1 that underwent PARylation procedure with PARP-1 but without NAD^+^). mTET1 incubated with PARP-1 without co-substrate NAD^+^ showed only a mild decrease in activity (on average by 25%), indicating that PARP-1 activity is required for the observed inhibitory effects. Moreover, neither NAD^+^ alone nor PARP-1 storage buffer had any effect on mTET1 activity. Overall, this experiment showed that PARylation by PARP-1 inhibits TET1 activity in vitro.

### TET1/PARP-1 cross-talk in cellulo

**Fig. 4:**
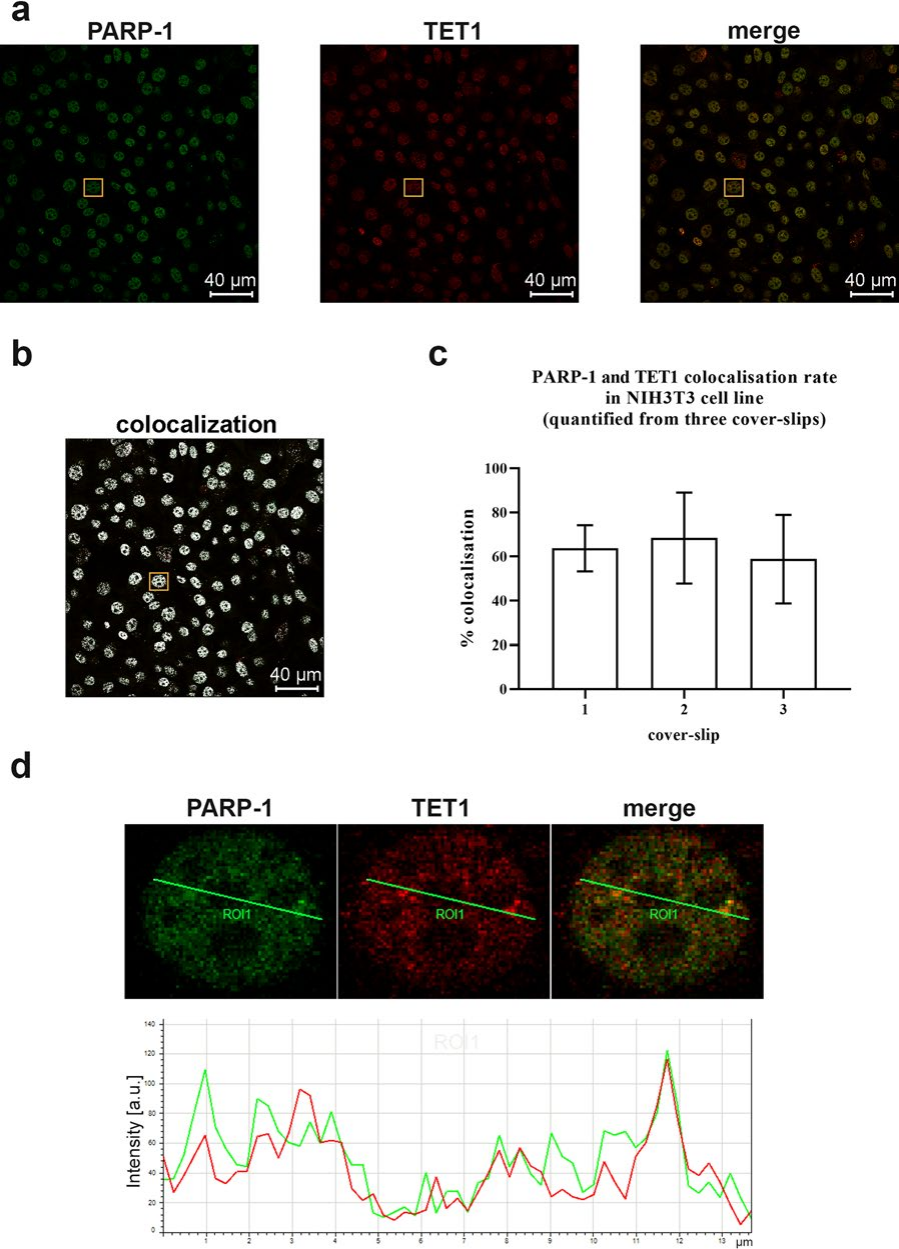
Colocalisation and co-immunoprecipitation of TET1 and PARP-1 proteins in NIH3T3 cells.** a** Confocal imaging of PARP-1 (green) and TET1 (red) stained by fluorescent secondary antibodies. **b** Colocalisation is evidenced by the merged confocal image and the image where sites of colocalisation (points where both PARP-1 and TET1 signals are detected) are highlighted in white. **c** Quantification of colocalisation rate from three replicate cover-slips analysed. For each cover-slip, 10 images were analysed and the colocalisation rate is shown as mean ± SD (n = 3). **d** Example of colocalisation in one enlarged nucleus (marked by □ in panels a and b). The intensity of green and red signals is measured along the green line (ROI1). n‐number of independent experiments, a.u.—arbitrary units, IP—immunoprecipitation, Ab—antibody, WB—western blot

Confocal microscopy of double fluorescently stained NIH3T3 cells showed that TET1 (labelled red) and PARP-1 (labelled green) proteins colocalise in the nucleus (Fig. 4a–c) with an average colocalisation rate of about 60%. In addition, on the example of a single cell, it was observed that, along a randomly selected line crossing the nucleus, the red and green signals follow a very similar profile (Fig. 4d), further confirming the colocalisation of TET1 and PARP-1 proteins in nuclei of NIH3T3 cells. The demonstrated colocalisation of TET1 and PARP-1 indicated that these proteins are located in close proximity in the nucleus and their interaction was also confirmed by immunoprecipitation (Additional file 1: Fig. S2).

### Inhibition of PARylation by niraparib

**Fig. 5:**
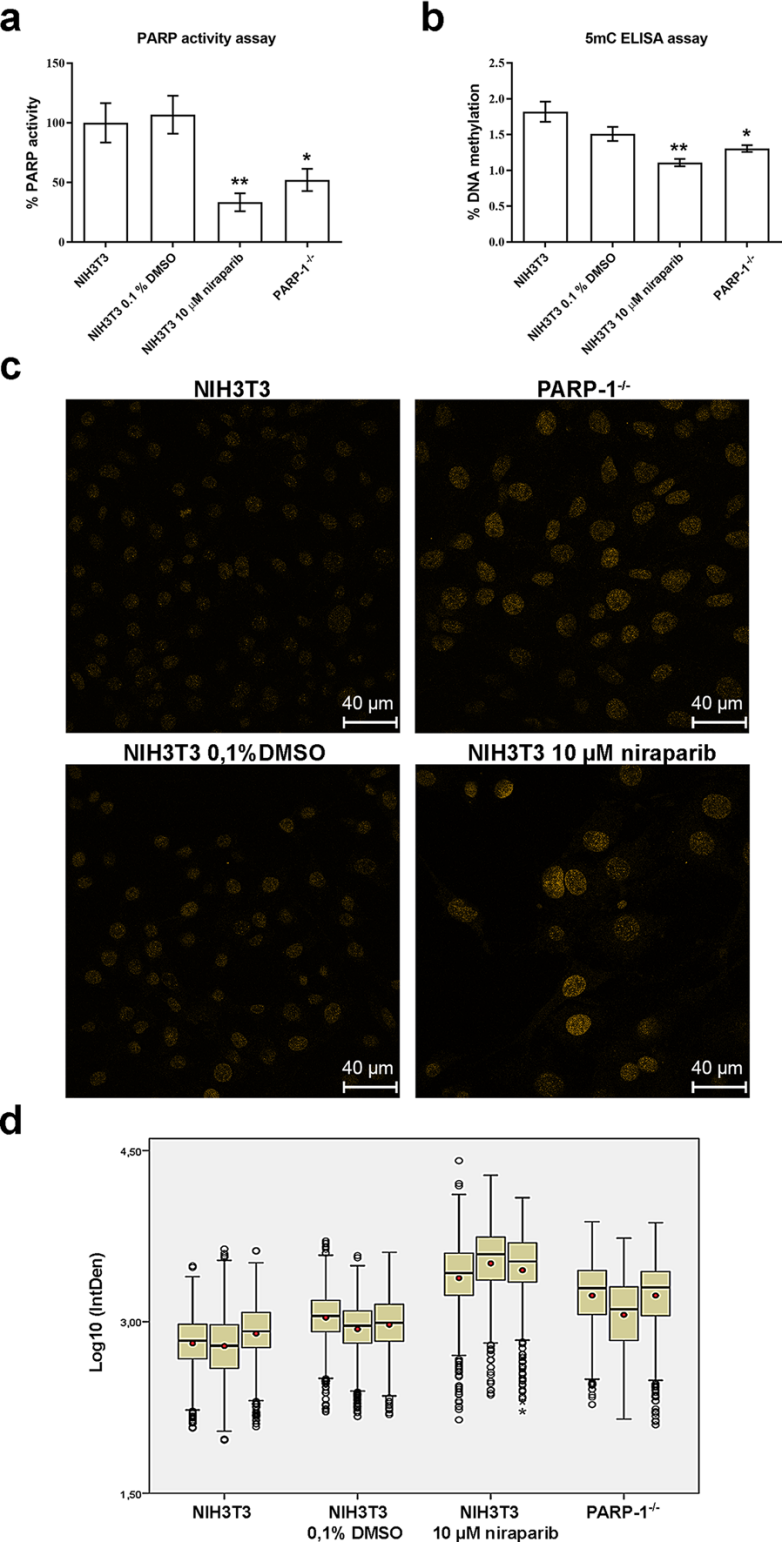
PARP activity, global DNA methylation and hydroxymethylation in niraparib treated NIH3T3 cells and in PARP^−/−^ cells.** a** PARP activity was evaluated by ELISA-based assay. Results were scaled to control NIH3T3 cells and are shown as mean ± SEM (n = 3). Statistical significance was evaluated by ANOVA (with blocking by sets of samples processed together) followed by a Dunnett test comparing each group to the control group. **b** Global level of DNA methylation was evaluated by an ELISA-based assay. Based on the absorbance measured for the standards, the calibration curve was approximated via a second-order logarithmic regression equation. The percentage of 5mC in the tested samples was calculated from the calibration curve. Results are presented as mean ± SEM (n = 3). Statistical significance was evaluated by ANOVA (with blocking by sets of samples processed together) followed by a Dunnett test comparing each group to the control group. **c** Immunocytological detection of 5hmC, with anti-5hmC antibody, by confocal imaging. **d** Quantification of 5hmC signal in confocal images. Integrated signal density (IntDen) of single nuclei was Log10 transformed and represented by a box-plot and the mean value for each sample was marked (●). Statistical significance was evaluated by nested ANOVA followed by the Tukey post hoc test. All groups are significantly different from each other at p*** ≤ 0.001. *p ≤ 0.05, **p ≤ 0.01, n‐number of independent experiments

The level of PARP activity in lysates, obtained from control NIH3T3 cells, NIH3T3 cells treated with niraparib or DMSO as well as from PARP-1^−/−^ cells, was determined by PARP activity assay (Fig. 5a). As expected, upon treatment with 10 μM niraparib, the observed PARP activity was decreased in treated NIH3T3 cells to 33.4% (p = 0.009) and in PARP-1^−/−^ cells to 52.1% (p = 0.039) while in NIH3T3 cells treated with DMSO no significant change was observed when compared to NIH3T3 controls (Fig. 5a).

### Influence of PARylation on global DNA (de)methylation

We measured the global DNA methylation levels, reflected as the levels of 5mC, by ELISA-based assay, in control NIH3T3 cells, NIH3T3 cells treated with niraparib or DMSO as well as in PARP-1^−/−^ cells (Fig. 5b). A statistically significant decrease in global methylation levels was detected in NIH3T3 cells treated with 10 μM niraparib (p = 0.008) as well as in PARP-1^−/−^ cells (p = 0.034) compared to control NIH3T3 cells, while DMSO treatment did not lead to a significant change. The average level of 5mC in control NIH3T3 cells was 1.82%, while in NIH3T3 cells treated with niraparib it was reduced to 1.12% and in PARP-1^−/−^ cells to 1.30%. These results indicated that the absence of PARP-1, as well as the inhibition of PARylation, leads to a decrease in the global level of DNA methylation in cellulo with inhibition of PARP activity having a more pronounced effect.

### Influence of PARylation on global DNA hydroxymethylation

To determine whether the decreased level of 5mC upon inhibition of PARylation was due to the increased TET activity, the global level of the 5hmC was examined. Hydroxymethylation of DNA was evaluated by confocal fluorescence microscopy of 5hmC stained NIH3T3 cells, niraparib- or DMSO-treated NIH3T3 cells as well as PARP-1^−/−^ cells (Fig. 5c). The specificity of staining was confirmed by the detection of the increased signal after treatment of NIH3T3 cells by ascorbic acid, known to induce TET activity (Additional file 1: Fig. S4). For more accurate comparisons, the immunofluorescence signal of 5hmC was quantified and represented as the integrated signal density of a nucleus (Fig. 5d). Three microscopic slides were prepared for each group of cells and a total of 2574 nuclei of control NIH3T3 cells, 2899 nuclei of NIH3T3 cells treated with 0.1% DMSO, 1032 nuclei of NIH3T3 cells treated with 10 μM niraparib and 2009 nuclei of PARP-1^−/−^ cells were quantified. All groups showed statistically significant differences at p ≤ 0.001 (Additional file 2: Table S1). After quantification by Image J software the highest level of 5hmC was detected in NIH3T3 cells treated with 10 μM niraparib, followed by PARP-1^−/−^ and DMSO-treated NIH3T3 cells, while the weakest signal was detected in control NIH3T3 cells. There is evidence that DMSO treatment may affect 5hmC levels [28] which was also observed in this experiment. However, treatment with niraparib (dissolved in DMSO) led to a significantly larger increase in 5hmC signals, which unequivocally indicate that inhibition of PARylation positively affects DNA hydroxymethylation. The effect of niraparib treatment was also confirmed by slot-blot (Additional file 1: Fig. S5). These results suggest an inhibitory effect of PARylation on TET-mediated DNA demethylation in cellulo.

## Discussion

Previously, we have discovered a connection between PARP-1 and TET enzymes on a local genomic level. We observed the inhibitory effect of PARP-1 on the demethylation of the *Cxcl12* gene [20, 21]. This study further explored the influence of PARP-dependent PARylation on TET hydroxylase activity at the genome level.

Consistent with a previous observation [17], we show that the recombinant murine TET1 catalytic domain is PARylated by PARP-1 in vitro. Furthermore, we show that the catalytic domain of TET2 is also PARylated in vitro, which may indicate the functional significance of already established PARP-1/TET2 interaction [19, 21]. To enable purification of the TET2 catalytic domain, used in our in vitro experiments, its long unstructured region had to be removed [29], thus implying that PARylation target sites are within the structured parts of the TET2 catalytic domain. In patients with leukaemia, the most common point mutations of human TET2 lead to disruption of its catalytic activity and are grouped within well-structured parts of the catalytic domain, on its surface [30, 31]. It is assumed that the side groups of these surface amino acids, can participate in protein–protein interactions and be target sites of posttranslational modifications [31]. We also examined the ability of PARP-2 to PARylate TET1 and TET2, as it has some overlapping roles with PARP-1 [32, 33], showing for the first time that it can indeed modify both TET1 and TET2 in vitro*.*

PARylation can alter the electrostatic and topological characteristics of modified proteins and affect their catalytic activities [34]. Due to complex interplay, the influence that PARylation exerts on TET activity has not yet been unambiguously determined. The complexity of the influence of PARylation on the methylation status was highlighted in a study where inhibition of PARylation in the MCF7 cell line led to hypermethylation in 72% and hypomethylation in 28% of the analysed loci, although the total global level of methylation remained unchanged [35]. A positive association of PAR levels with 5hmC and 5fC has been recently found in the genome of patients affected by type 2 diabetes mellitus [36]. Similarly, in zebrafish with induced diabetes mellitus, the inhibition of PARylation neutralised the increase of 5hmC, yet did not affect the 5hmC level in healthy animals [37], suggesting that PARylation may have varying effects within the same organism depending on conditions. In yet another study, it was observed that inhibition of PARylation leads to a decrease in 5hmC levels of HEK239T cells while the nuclear lysate of these cells, in an in vitro assay, shows enhanced hydroxylation of the methylated DNA substrate, after the same treatment [17]. In addition, in vitro experiments have shown that PARylation of the recombinant catalytic domain of human TET1 leads to stimulation of its activity while non-covalent interaction with PAR polymers has the opposite, inhibitory effect [17]. Non-covalent interaction with PAR polymers also serves to recruit TET proteins to certain sites in chromatin [18], which can further complicate the determination of the influence of PARylation on TET enzymatic activity. However, in the experiments with overexpression of engineered TET1 fused to specific DNA binding domains, inhibition of PARylation resulted in an increased global level of 5hmC that was exclusively due to altered TET1 activity rather than its recruitment by PAR polymers [17]. From this, the authors inferred that PARylation has an inhibitory effect on the enzymatic activity of TET1 protein in vivo [13, 17]. Consistently, our results have shown that PARylation led to decreased TET1 enzymatic activity evidenced by lower initial oxidation rates of 5mC to 5hmC by TET1 in vitro. The increase in PARylation levels, caused by the increased concentration of PARP-1, led to a progressive decline in TET1 activity which further confirms the inhibitory role of this posttranslational modification on TET1 hydroxylase activity. It should be noted that in vitro PARylation of TET1 does not eliminate the possibility of non-covalent interaction with PAR polymers, which may also contribute to the overall effect. Accordingly, the intertwining of covalent TET1 PARylation and non-covalent interaction with PAR polymers can also be expected to occur in vivo.

To gain insight into the functional implications of the PARylation of TET proteins in cellulo, the experiments were performed on the NIH3T3 cell line starting from the analysis of PARP-1/TET1 colocalisation and mutual protein/protein interactions to the assessment of global DNA (de)methylation status upon PARP inhibition. Colocalisation of TET1 and PARP-1 in the nucleus, as well as their mutual co-immunoprecipitation clearly shows that TET1 and PARP-1 proteins interact in the nuclei of NIH3T3 cells. Accordingly, another study showed that recombinant TET1 protein expressed in HEK293T cells interacts with endogenous PARP-1, as well as that all three members of the TET family, expressed in recombinant form, colocalise and interact with recombinant PARP-1 protein [19]. Besides, the interaction of endogenous TET1 and PARP-1 has been detected in HEK293T cells and it has been confirmed in vitro that this interaction can be established directly, without the mediation of other proteins [17]. In addition, we have previously demonstrated that TET2 can likewise interact with PARP-1 in NIH3T3 cells [21].

Based on the observed decreased 5mC and increased 5hmC levels in NIH3T3 cells treated with niraparib and in PARP-1^−/−^ cells, we provide further evidence for the inhibitory effects of PARP-1 and PARylation on TET hydroxylase activity in cellulo. The inhibition of PARP activity and the absence of PARP-1 affect TET hydroxylase activity to a different extent, showing a more pronounced inhibition in cells treated with PARP inhibitor than in PARP1^−/−^ cells. Although the level of PARylation is noticeably reduced in PARP-1^−/−^ cells, it suggests that PARP-2 present in the cells can compensate the lack of PARP-1 by PARylating TET1 [32, 33]. PARP-2 is known to be less abundant and contributes between 5 and 10% of the total PARP activity in the cells [38, 39]. Bearing in mind that we have here shown that PARP-2 can PARylate TET proteins in vitro and that it has been implicated in the regulation of TET1 gene expression [15], it is possible that PARP-2 could, to some extent, neutralise the increase of 5hmC resulting from the lack of PARP-1, in PARP-1^−/−^ cells. The catalytic centre of PARPs is highly conserved, and niraparib (like most other PARP inhibitors) is not selective and efficiently inhibits both PARP-1 and PARP-2 [40]. Consistently with our observations, the more pronounced 5hmC increase, detected after treatment of NIH3T3 cells with niraparib, can be attributed to the inhibition of both PARP proteins' activity.

Though PARP-1 and PARylation can influence DNA (de)methylation by modulating DNA methyltransferase expression and activity [14, 41], a detected increase in hydroxymethylation indicates that in this study DNA demethylation is achieved via TET activity induced by the lack of PARP-1 or inhibited PARylation. This is in line with and expands on our previous research showing that the inhibitory role of PARP-1 in the local regulation of *Cxcl12* gene expression is in part achieved via the negative influence of PARP-1 on local TET-mediated DNA demethylation [20, 21]. The activating effects on TET activity shown in our study could be the base for additional evaluation of the efficacy of PARP inhibitors in the treatment of cancers that are characterised by the diminishing level of 5hmC.

## Conclusion

Taken together, the findings presented in this study strongly support the inhibitory influence of PARP-1-dependent PARylation on TET1 hydroxylase activity in DNA demethylation at the global genome level. It makes biological sense that a protein involved in DNA repair, such as PARP-1, also has the ability to inhibit TET activity, in order to protect genome stability by preventing the excessive generation of DNA repair intermediates formed in the process of active DNA demethylation. PARP-1 may therefore have a dual influence on DNA demethylation, on one hand, helping in completing the final steps of this process through involvement in BER and, on the other, targeting the beginning of the process by directly inhibiting TET activity through PARylation. This can be particularly useful in developing cancer treatments where PARP inhibition could at the same time harm cancer cells’ ability to repair DNA (synthetic lethality phenomenon) but also induce TET activity which can in turn propel the formation of abasic sites and genome destabilisation, leading to the cytotoxicity of cancer cells.

## Material and methods

### Expression and purification of recombinant proteins

The recombinant, histidine-tagged, proteins -catalytic domains (CD) of murine TET1 (1367–2038 aa) and TET2 (1044–1920 aa with the unstructured region replaced by a flexible 15aa linker) and the full-length murine PARP-1, were expressed in *Escherichia coli* BL21 (DE3) CodonPlus RIL (Novagen) cultivated in Luria–Bertani media. Protein expression was induced by 0.5 mM isopropyl β-D-1-thiogalactopyranoside and cultivation proceeded for 14–15 h at 20 °C. Harvested cells were washed with sodium chloride–Tris–EDTA buffer and resuspended in sonication/wash buffer (50 mM HEPES pH 6.8, 35 mM imidazole, 1 mM DTT, 500 mM NaCl, 10% glycerol) supplemented with protease inhibitor cocktail. Crude cell lysates were prepared by sonification, proteins were purified by Ni–NTA affinity chromatography (Genaxxon) and eluted with a buffer containing high imidazole concentration (50 mM Hepes pH 6.8, 300 mM imidazole, 1 mM DTT, 500 mM NaCl, 10% glycerol). Elution fractions with the highest protein concentrations were pulled together and dialysed for 3 h in dialysis buffer (50 mM Hepes pH 6.8, 1 mM DTT, 300 mM NaCl, 10% glycerol). Aliquots of purified recombinant proteins were flash-frozen in liquid nitrogen and stored at −80 °C until use.

### In vitro PARylation

In vitro PARylation was achieved by incubating 5 μM purified recombinant murine TET1-CD or TET2-CD with 0.18 μg of commercial human recombinant PARP-1 or PARP-2 (Enzo Life Sciences, Inc.) in 50 mM Tris–HCl pH 8, 1.5 mM DTT, 1 mM MgCl_2_, 0.25 μg/μl DNK salmon sperm, 200 μM NAD^+^ at room temperature (RT). The reaction was stopped after 5 min, 15 min, 30 min or 60 min by adding sample buffer. In the case of TET2 in vitro PARylation, one of each PARP-1 and PARP-2 reactions were stopped after 60 min by 8 μM niraparib (MedChemExpress) and then PARG enzyme (0.043 ng/ml) (Trevigen) was added and incubated at 37 °C for 1 h.

All samples were separated by Tris–glycine SDS-PAGE on 10% polyacrylamide gels and electro-transferred onto nitrocellulose membranes. Membranes were stained by Ponceau-S, photographed and probed by primary murine anti-PAR (1:1000, Enzo Life Sciences, Inc., H10) or a murine anti-HIS (1:2000, Roche) antibody and secondary anti-mouse horseradish peroxidase-conjugated secondary antibody (1:10,000, GE Health care). Signal was visualised on X-ray film with the enhanced chemiluminescence solution reagent (Thermo Scientific).

### ATP docking (SwissDock)

To identify possible adenine binding sits on the surface of the TET2 protein, we have used SwissDock platform. The structure of human TET2 catalytic domain was used as the template to dock ATP models. The resulting docking model was visualised in PyMol.

### ELISA-based plate assay for DNA hydroxymethylation analysis

Kinetics of TET activity was measured as previously described [27, 29], with minor modifications, by an ELISA-based assay that allows detection of 5hmC. Prior to the assay recombinant TET1-CD (1 μM) was in vitro PARylated for 5 min at RT with increasing concentrations of recombinant purified PARP-1 in a reaction mixture with biotin-labelled methylated DNA substrate. After TET activation reaction temperature was raised to 37 °C. At specific time points (0 s, 30 s, 1 min, 2 min, 3 min, 5 min, 7.5 min, 10 min) 2-μl aliquots were transferred into avidin-coated (Sigma-Aldrich) ELISA plate wells filled with NaOH to stop the reaction. After blocking with 2% BSA (Roth), primary rabbit anti-5hmC (1: 10,000, Active Motif) and subsequently secondary goat anti-rabbit HRP-conjugated (1: 5000, GE Healthcare) antibodies were added. The signal was developed using an ECL reagent (Thermo Scientific) and detected on a 2300 EnSpire Multimode ELISA reader (Perkin Elmer). Detailed protocol is presented in Additional file 3: materials and methods.

### Cell culture and treatment

Murine embryonic fibroblasts NIH3T3 (ATCC-CRL-1658) and embryonic fibroblasts isolated from PARP-1 knock-out mice[42] were cultivated in modified Dulbecco's modified Eagle medium (DMEM) (Biological Industries) to which penicillin, streptomycin and 10% fetal bovine serum were added. The cells were cultivated at 37 °C with 5% CO2 in the atmosphere and after reaching about 80% confluency, were subcultured by trypsinisation.

NIH3T3 cells were seeded in sterile 6-well plates and treated with 10 μM niraparib for 72 h (with treatment renewal every 24 h). Since niraparib was dissolved in dimethyl sulfoxide (DMSO), cells were also treated with DMSO in the final concentration of 0.1% as respective control.

### MTT assay

Cell viability was examined by 3-(4,5-dimethylthiazol-2-yl)-2,5-diphenyltetrazolium bromide (MTT) assay. Cells were seeded in 96-well plates. At 60–70% confluency cells were treated with niraparib (1.25 µM, 2.5 µM, 5 µM, 10 µM, 20 µM). After 72 h medium was replaced by 200 μl of 0.5 mg/ml MTT (Sigma, M5655) dissolved in DMEM. Following 2 h incubation at 37 °C, MTT was replaced by DMSO (100 µl/well). The absorbance was measured at 570 nm using an ELISA plate reader.

### Detection of protein colocalisation by immunocytochemistry

NIH3T3 cells were seeded on glass cover-slips (2 × 10^4^ cells per cover-slip) and were grown for 72 h. Cover-slips were washed with PBS, fixed with 2% paraformaldehyde (PFA) for 10 min at RT and cell permeabilisation was performed with 0.25% Triton X-100 at RT for 10 min. After blocking in 3% BSA cover-slips were incubated with rabbit anti-TET1 (1:10,000, Merck Millipore) followed by goat anti-rabbit Alexa Fluor 647 (1:400, Invitrogen) antibody. Thereafter, the cover-slips were incubated with rat anti-PARP-1 (1:25, R&D Systems) followed by secondary anti-rat FITC (1:400) antibody. After washes with PBST and water, the cover-slips were mounted on microscope slides with Mowiol reagent (Calbiochem).

Cover-slips were photographed on a Leica TCS SP5 II confocal microscope (laser excitation at wavelengths of 488 nm and 633 nm, × 63 magnification). Colocalisation analysis was done using LAS AF software, which sets thresholds for background and red and green fluorescent signals to determine points of colocalisation in the foreground area of the image. This software calculates colocalisation rate as the ratio of total image area where the overlapping of two colours (signals from two fluorophores) is detected, to the foreground area of the image. Same setup for signal and background thresholds was applied for analysis of all images.

### Determination of 5hmC level by immunocytochemistry

NIH3T3 cells were seeded on glass cover-slips (2 × 10^4^ cells per cover-slip) and were grown for 72 h. Cover-slips were washed with PBS, fixed with 2% PFA for 10 min at RT and cell permeabilisation was performed with 0.25% Triton X-100 at RT for 10 min. For DNA denaturation the cover-slips were incubated in 2 N HCl for 30 min at 37 °C. After blocking in 3% BSA cover-slips were incubated with a rabbit anti-5hmC (1:10,000, Active Motif) followed by secondary donkey anti-rabbit Alexa Fluor 555 (1:400, Invitrogen) antibody. After washes with PBST and water, the cover-slips were mounted on microscope slides with Mowiol reagent (Calbiochem).

Cover-slips were photographed on a Leica TCS SP5 II confocal microscope (laser excitation at the wavelength of 543 nm, × 63 magnification). Level of 5hmC was analysed using ImageJ 1.52p software [43, 44] with a macro written for the analysis (Additional file 3: Materials and methods).

### PARP activity assay

PARP Universal Colorimetric Assay Kit (Trevigen) was used according to the manufacturer's instructions Histone-coated wells were filled with 25 μl of lysate containing 40 μg of protein and PARP buffer. This ELISA-based assay measures incorporation of biotinylated PAR onto histone proteins after addition of PARP cocktail with biotinylated NAD^+^. The absorbance was measured at 450 nm using an ELISA plate reader.

### Genomic DNA isolation and 5mC DNA ELISA assay

Cells were lysed overnight at 55 °C with modified Bradley buffer (2 mM EDTA, 10 mM NaCl, 0.5% SDS, 10 mM Tris–HCl, pH 7.5) supplemented with proteinase K. DNA was isolated by ethanol precipitation (using absolute ethanol supplemented with 75 mM Na-acetate followed by washes in 70% ethanol) and dissolved in water.

Global DNA methylation levels were measured using a commercial 5mC DNA ELISA Kit (Zymo Research) according to the manufacturer's instructions. The wells were coated with denatured DNA (100 ng genomic DNA and methylated DNA standards) and methylation was detected after addition of anti-5mC and HRP-conjugated secondary antibody mixture. The absorbance was measured at 450 nm using an ELISA plate reader.

### Statistical analysis

For PARP activity assay and 5mC DNA ELISA assay, statistical significance was analysed by the one-way ANOVA test with blocking, where the set of results of all groups of one independent experiment represented a block. The result of each experimental group was compared with the results of the control group of NIH3T3 cells by Dunnett’s test.

For determination of 5hmC level by immunocytochemistry results were log10 transformed and statistical significance was analysed by nested ANOVA followed by Tukey post hoc test.

Statistical analysis and graphical representation of results were done using IBM SPSS Statistics 20.0, GraphPad Prism 8.0.2 and Microsoft Excel 2013 (Microsoft Corp.).

## Supplementary Information

**Additional file 1: Figures S1**. I*n vitro* PARylation of TETs. Figure S2. Co-immunoprecipitation of TET1 and PARP-1 from NIH3T3 cell lysates. Figure S3. MTT viability assay. Supplementary Figure S4. Level of 5hmC in control and ascorbic acid treated NIH3T3cells.

**Additional file 2: Table S1.** Statistical significance of 5hmC level differences between groups evaluated by nested ANOVA followed by Tukey post hoc test.

**Additional file 3: **Materials and methods: ELISA-based plate assay for DNA hydroxymethylation analysis, determination of 5hmC level by immunocytochemistry and co-immunoprecipitation.

## Data Availability

All data generated or analysed during this study are included in this published article and its additional information files.

## Abbreviations

PARP-1: Poly(ADP-ribose) polymerase-1
PARylation: Poly(ADP-ribosyl)ation
PAR: Poly(ADP-ribose)
BER: Base excision repair
5hmC: 5-Hydroxymethylcytosine
5fC: 5-Formylcytosine
5caC: 5-Carboxylcytosine
TET: Ten-eleven translocation
DMSO: Dimethyl sulfoxide
