# Supplementary material for: TET-mediated DNA hydroxymethylation is negatively influenced by the PARP-dependent PARylation
Source: Epigenetics Chromatin. 2022 Apr 5;15:11. doi: 10.1186/s13072-022-00445-8 (PMC8985375; doi:10.1186/s13072-022-00445-8)
Supplement: Supplementary file 1 — Additional file 1: Figures S1. In vitro PARylation of TETs. Figure S2. Co-immunoprecipitation of TET1 and PARP-1 from NIH3T3 cell lysates. Figure S3. MTT viability assay. Supplementary Figure S4. Level of 5hmC in control and ascorbic acid treated NIH3T3cells. [file 13072_2022_445_MOESM1_ESM.docx]

**Additional file 1**

**Supplementary Figures**

**
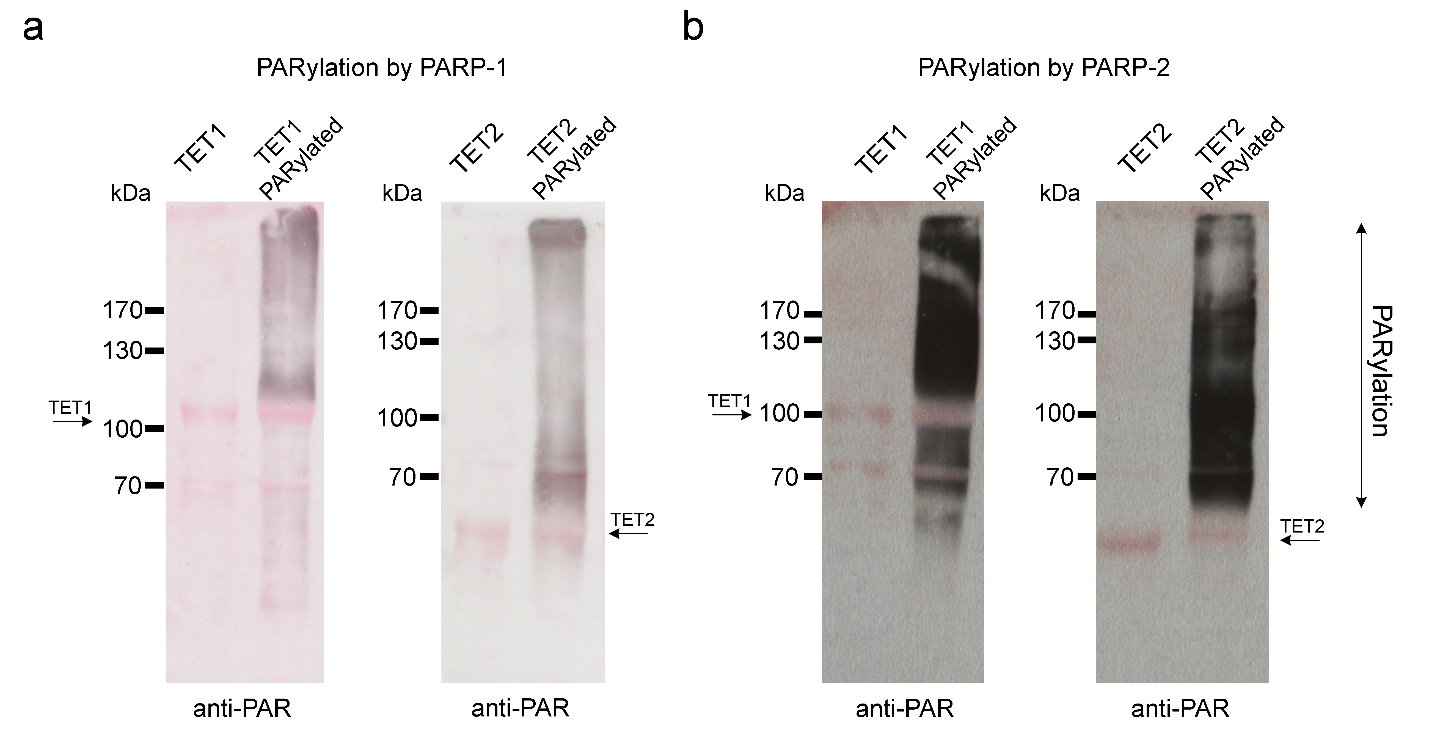
**

**Supplementary Figure S1. *In vitro* PARylation of TETs.** TET1 and TET2 were *in vitro* PARylated by **(a)** PARP-1 and **(b)** PARP-2 and separated by tris-glycine SDS-PAGE. Images of Ponceau S stained membranes were merged with immunoblots stained with anti-PAR antibody.

**
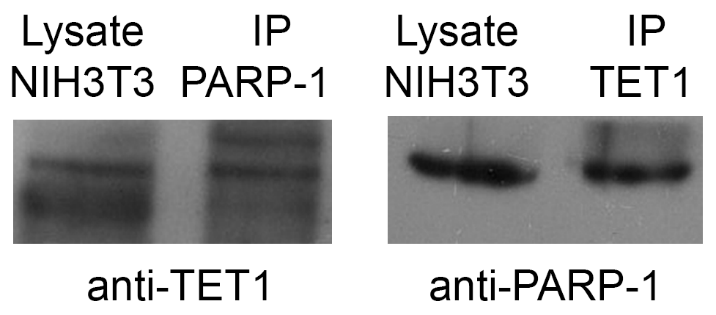
**

**Supplementary Figure S2. Co-immunoprecipitation of TET1 and PARP-1 from NIH3T3 cell lysates.** Immunoblot detection of TET1 and PARP-1 proteins in immunoprecipitates by anti-TET1 and anti-PARP-1antibodies. IP – immunoprecipitation.

**
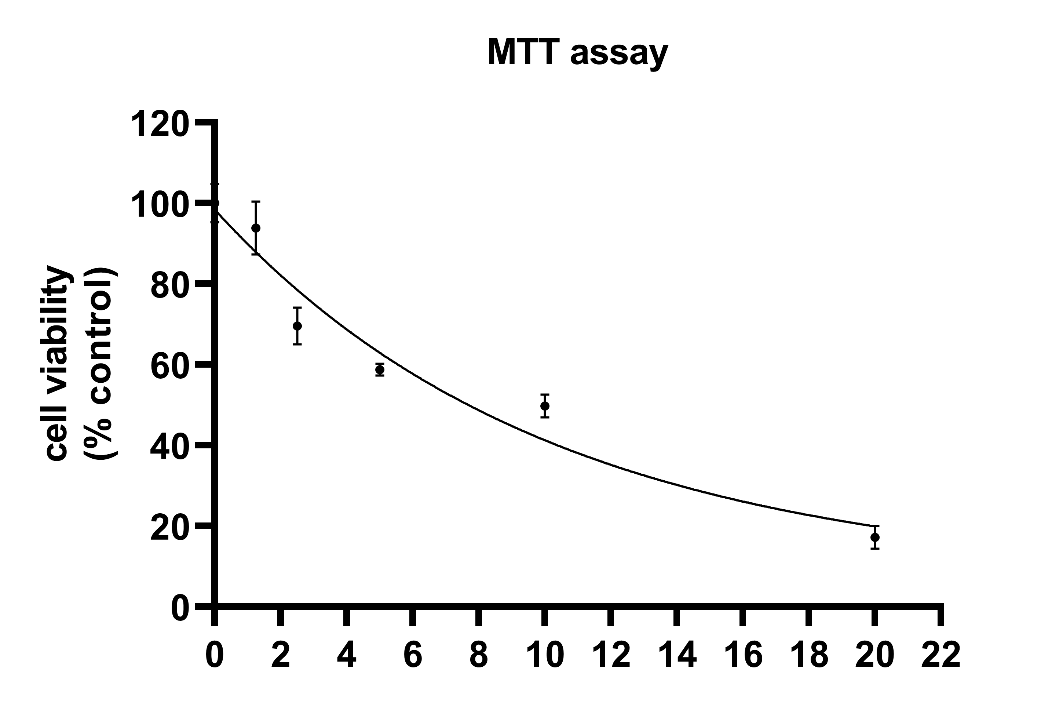
**

**Supplementary Figure S3. MTT viability assay.** The viability of NIH3T3 cells treated with increasing concentrations of PARylation inhibitor niraparib was evaluated by MTT assay. Data points are presented as mean ± SEM (n = 3). n‐number of independent experiments.

**
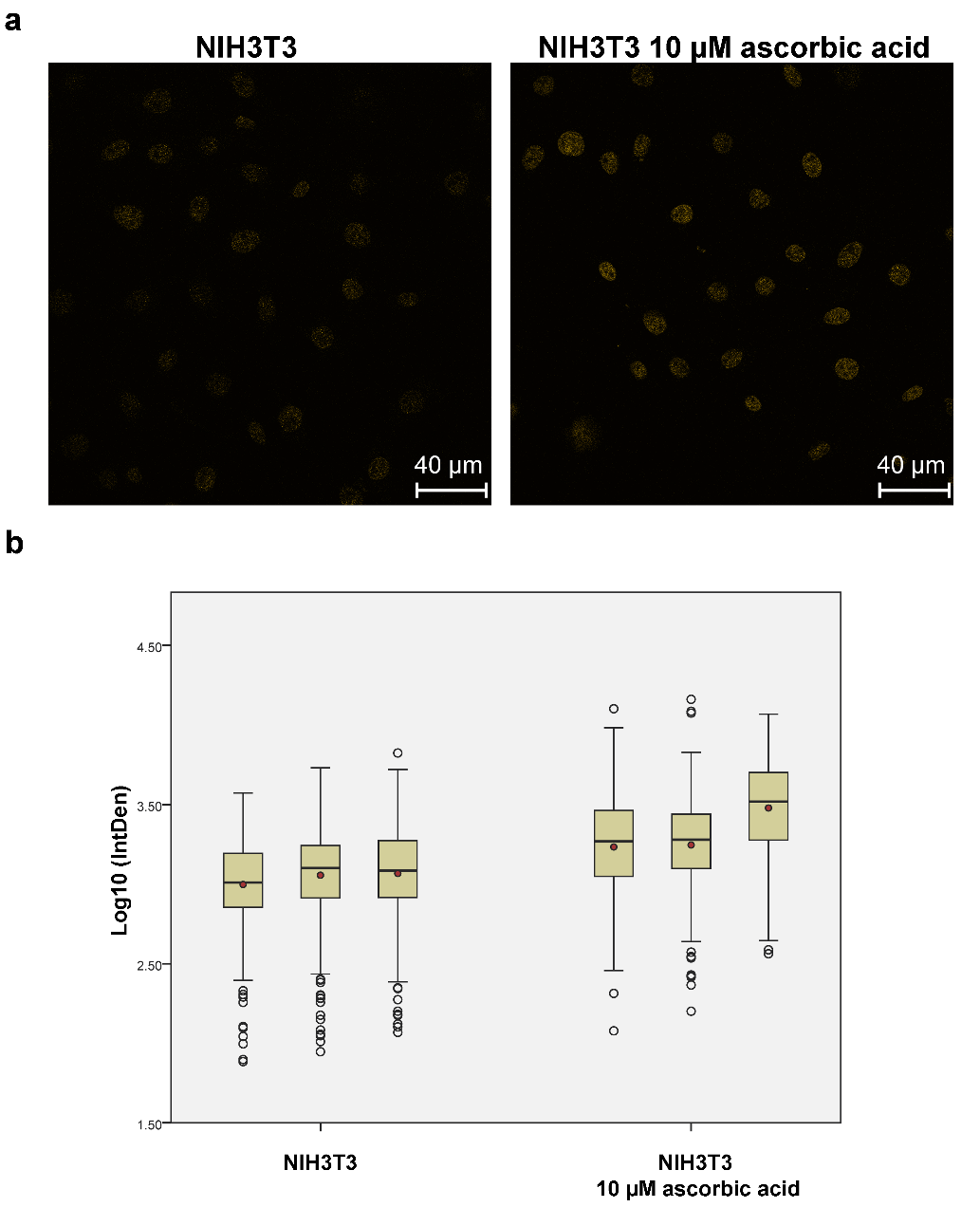
**

**Supplementary Figure S4. Level of 5hmC in control and ascorbic acid treated NIH3T3cells.** **(a)** Immunocytological detection of 5hmC, with anti-5hmC antibody, by confocal imaging. **(b)** Quantification of 5hmC signal in confocal images. Three microscopic slides were prepared for each group of cells and a total of 1122 nuclei of control NIH3T3 cells and 743 nuclei of NIH3T3 cells treated with 10 μM ascorbic acid were quantified. Integrated signal density (IntDen) of single nuclei was Log10 transformed and represented by a box-plot and the mean value for each sample was marked (●). Statistical significance was evaluated by nested ANOVA. There is a significant difference between control and ascorbic acid treated NIH3T3 cells at the p* ≤ 0.05.

**
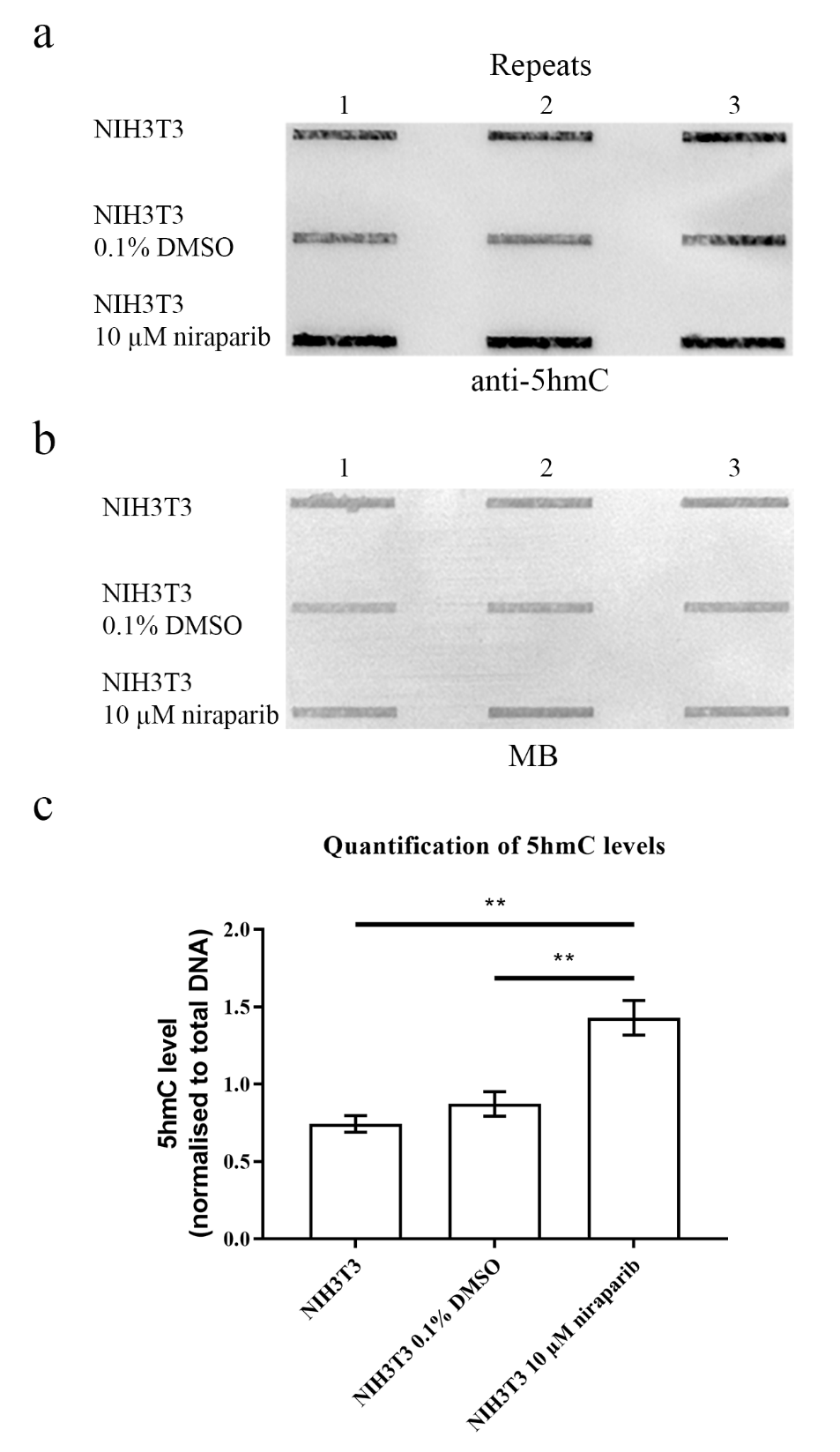
**

**Supplementary Figure S5. Level of 5hmC in niraparib treated NIH3T3 cells detected by slot-blot. (a)** Slot-blot detection of 5hmC in NIH3T3 DNA probed with anti-5hmC antibody. **(b)** Slot-blot detection of total DNA by methylene blue (MB) staining. **(c)** Quantification of 5hmC levels normalized to total DNA as measured by MB staining. Results are presented as mean ± SEM (n = 3). Statistical significance was evaluated by ANOVA followed by the Tukey Post Hoc test. **p ≤ 0.01
