# Supplementary material for: TET-mediated DNA hydroxymethylation is negatively influenced by the PARP-dependent PARylation
Source: Epigenetics Chromatin. 2022 Apr 5;15:11. doi: 10.1186/s13072-022-00445-8 (PMC8985375; doi:10.1186/s13072-022-00445-8)
Supplement: Supplementary file 2 — Additional file 2: Table S1. Statistical significance of 5hmC level differences between groups evaluated by nested ANOVA followed by Tukey post hoc test. [file 13072_2022_445_MOESM2_ESM.docx]

**Additional file 2**

**Supplementary Table**

|  | 5hmC level compared to | p= |
| --- | --- | --- |
| NIH3T3 control | NIH3T3 0.1% DMSO | 5.492E-13 |
|  | NIH3T3 10 µM niraparib | 5.492E-13 |
|  | PARP-1^-/-^ | 5.492E-13 |
| NIH3T3 0.1% DMSO | NIH3T3 control | 5.492E-13 |
|  | NIH3T3 10 µM niraparib | 5.492E-13 |
|  | PARP-1^-/-^ | 5.492E-13 |
| NIH3T3 10 µM niraparib | NIH3T3 control | 5.492E-13 |
|  | NIH3T3 0.1% DMSO | 5.492E-13 |
|  | PARP-1^-/-^ | 5.492E-13 |
| PARP-1^-/-^ | NIH3T3 control | 5.492E-13 |
|  | NIH3T3 0.1% DMSO | 5.492E-13 |
|  | NIH3T3 10 µM niraparib | 5.492E-13 |

**Supplementary Table 1.** Statistical significance of 5hmC level differences between groups evaluated by nested ANOVA followed by Tukey Post Hoc test.

|  |  |  | TET1+PARP-1+NAD^+^ | | | | |  |
| --- | --- | --- | --- | --- | --- | --- | --- | --- |
| 1st repeat time [min] | TET1 control | TET1 (-PARP-1) +NAD^+^ | 0.125µM PARP-1 | 0.25µM PARP-1 | 0.5µM PARP-1 | 1µM PARP-1 | 2µM PARP-1 |  |
| 0 | 163481 | 33629 | 30984 | 31152 | 28817 | 41266 | 62107 |  |
| 0.5 | 409511 | 90664 | 60915 | 66652 | 46621 | 72476 | 47629 |  |
| 1 | 1439462 | 516998 | 269981 | 214733 | 120596 | 102831 | 117532 |  |
| 2 | 4166179 | 2788572 | 1847263 | 855902 | 377395 | 256773 | 299146 |  |
| 3 | 5113331 | 4294629 | 2797972 | 1862493 | 659176 | 392921 | 437225 |  |
| 5 | 5528164 | 5874924 | 4843088 | 3363172 | 1332850 | 852821 | 1438168 |  |
| 7.5 | 6419666 | 7197141 | 5750271 | 4414708 | 2060301 | 1691847 | 2195345 |  |
| 10 | 6393310 | 6894714 | 5908671 | 5158013 | 2710648 | 2485688 | 3306418 |  |
|  |  |  |  |  |  |  |  |  |
|  |  |  |  |  |  |  |  |  |
|  |  |  | TET1+PARP-1+NAD^+^ | | | | |  |
| 2nd repeat time [min] | TET1 control | TET1 (-PARP-1) +NAD^+^ | 0.125µM PARP-1 | 0.25µM PARP-1 | 0.5µM PARP-1 | 1µM PARP-1 | 2µM PARP-1 |  |
| 0 | 58339 | 23147 | 25842 | 18155 | 23071 | 22430 | 27093 |  |
| 0.5 | 308725 | 33918 | 36541 | 31403 | 28090 | 23534 | 32441 |  |
| 1 | 955630 | 247695 | 115079 | 88560 | 69109 | 27810 | 33763 |  |
| 2 | 2314062 | 1326381 | 786538 | 552374 | 365277 | 72901 | 97555 |  |
| 3 | 4163724 | 2719332 | 1613564 | 1267253 | 940730 | 240076 | 329560 |  |
| 5 | 6111034 | 3358090 | 3144637 | 2591595 | 1971896 | 684180 | 914547 |  |
| 7.5 | 6570813 | 4198000 | 4102030 | 3701915 | 3143766 | 1330921 | 1855992 |  |
| 10 | 7116106 | 5491948 | 4660540 | 4632806 | 3887898 | 1740117 | 2023141 |  |
|  |  |  |  |  |  |  |  |  |

| 1st repeat time [min] | TET1 control | TET1 (-PARP-1) +NAD^+^ | TET1 (-PARP-1 -NAD^+^) | TET1+2µM PARP-1 (-NAD^+^) | TET1+2µM PARP-1 +NAD^+^ |
| --- | --- | --- | --- | --- | --- |
| 0 | 78465 | 179011 | 277618 | 66744 | 69780 |
| 0.5 | 287168 | 250602 | 682664 | 97610 | 76412 |
| 1 | 934041 | 816152 | 1590515 | 423492 | 202364 |
| 2 | 3375366 | 3551633 | 4343540 | 1967052 | 911444 |
| 3 | 4815880 | 4632458 | 5278652 | 3194215 | 1640016 |
| 5 | 6053449 | 5830842 | 5799667 | 4358186 | 3079134 |
| 7.5 | 6711088 | 5887699 | 6154240 | 5085332 | 3679228 |
| 10 | 6300847 | 7023461 | 6896054 | 5734619 | 4802336 |
|  |  |  |  |  |  |
|  |  |  |  |  |  |
| 2nd repeat time [min] | TET1 control | TET1 (-PARP-1) +NAD^+^ | TET1 (-PARP-1 -NAD^+^) | TET1+2µM PARP-1(-NAD^+^) | TET1+2µM PARP-1+NAD^+^ |
| 0 | 54011 | 140994 | 133535 | 109585 | 21751 |
| 0.5 | 127259 | 260902 | 269944 | 177416 | 57576 |
| 1 | 806136 | 1229179 | 1081223 | 889958 | 222945 |
| 2 | 3994842 | 4310094 | 3172714 | 3293630 | 1013968 |
| 3 | 5672019 | 5703957 | 4249260 | 4874845 | 2370099 |
| 5 | 7362387 | 7095496 | 7642531 | 6407733 | 3785268 |
| 7.5 | 8309069 | 7942402 | 8289193 | 6620745 | 4476803 |
| 10 | 8011161 | 7712951 | 8649872 | 6395844 | 4771780 |

**Supplementary Tables 2.** Original data for kinetics of TET activity of *in vitro* PARylated TET1 (first two tables) and TET1 in control reactions (last two tables).
