# Supplementary material for: TET-mediated DNA hydroxymethylation is negatively influenced by the PARP-dependent PARylation
Source: Epigenetics Chromatin. 2022 Apr 5;15:11. doi: 10.1186/s13072-022-00445-8 (PMC8985375; doi:10.1186/s13072-022-00445-8)
Supplement: Supplementary file 3 — Additional file 3: Materials and methods: ELISA-based plate assay for DNA hydroxymethylation analysis, determination of 5hmC level by immunocytochemistry and co-immunoprecipitation. [file 13072_2022_445_MOESM3_ESM.docx]

**Additional file 3**

**Supplementary Materials and methods**

**ELISA-based plate assay for DNA hydroxymethylation analysis**

For the assay 86 bp biotinylated DNA substrate was used (amplified from a pUC19 vector by a primer pair DpnII_pUC19_f: 5’-GAGTAAACTTGGTCTGACAGTTACCA-3’ and DpnII_pUC19_r: 5’-CAACTATGGATGAACGAAATAGACAGAT-3’, with biotinylated forward primer). All cytosines in the substrate were methylated as the amplification reaction was done with 5mdCTP (New England Biolabs) instead of dCTP. The assay was performed in an avidin-coated (SigmaAldrich) 96-well plate. The plate was washed with PBST-500 buffer (PBS with 0.1% Tween supplemented with 500 mM NaCl) and then filled with 100 μl of 0.05 M NaOH. Recombinant TET1-CD (1 μM) was *in vitro* PARylated for 5 min at room temperature with increasing concentrations of recombinant purified PARP-1 in a reaction mixture containing 50 mM HEPES pH 6.8, 1.5 mM DTT, 400 ng methylated DNA substrate and 200 μM NAD^+^. Subsequently, the reagents necessary for TET activation (100 μM Fe^2+^, 1 mM αKG, 1 mM VitC, 150 mM NaCl) were added and the reaction temperature was raised to 37°C. At specific time points (0 s, 30 s, 1 min, 2 min, 3 min, 5 min, 7.5 min, 10 min) 2 μl aliquots (containing 40 ng of biotinylated DNA substrate) were transferred into plate wells filled with NaOH to stop the reaction. The biotinylated DNA substrate was incubated in avidin-coated wells for 1.5 h with shaking. After three washing with PBST-500, nonspecific antibody binding was blocked by 1 h incubation with 2% BSA (Roth) in PBST buffer. The next step was incubation with the primary rabbit anti-5hmC antibody (1:10000, Active Motif) for 1.5 h, followed by washing and incubation with goat anti-rabbit HRP-conjugated secondary antibody (1:5000, GE Healthcare) for 1 h. After rinsing, the signal was developed using an ECL reagent (Thermo Scientific) and detected on a 2300 EnSpire Multimode ELISA reader (Perkin Elmer).

**Determination of 5hmC level by immunocytochemistry**

Image J Macro written for the analysis of 5hmC level enabled automatic selection of individual nuclei in the images and measurement of fluorescence intensity expressed as the integrated signal density (IntDen) for each nucleus. The code for the macro used is shown here:

name = getTitle();

run("Duplicate...", " ");

dupl = getTitle(); selectWindow(name);

run("Subtract Background...", "rolling=50");

selectWindow(dupl);

run("Subtract Background...", "rolling=50");

run("Find Edges");

run("Gaussian Blur...", "sigma=3");

setAutoThreshold("Default dark");

setAutoThreshold("Yen dark");

setOption("BlackBackground", false);

run("Convert to Mask");

run("Fill Holes");

run("Watershed");

run("Set Measurements...", "area decimal=3");

run("Analyze Particles...", "size=25-Infinity circularity=0.5-1.00 show=Ellipses exclude include");

elips = getTitle();

run("Fill Holes");

run("Watershed");

run("Set Measurements...", "area mean standard modal min integrated median display redirect=["+ name +"] decimal=3");

run("Analyze Particles...", "size=25-Infinity circularity=0.50-1.00 show=Overlay display exclude include");

dir = getDirectory("Choose the directory to save results to");

saveAs("Measurements", dir+name+"YenResults.csv");

run("Clear Results");

**Co-immunoprecipitation**

Cell lysates were isolated by ProteoJET Mammalian Cell Lysis Reagent (Thermo Scientific), according to the manufacturer's instructions. Lysates (300 µg) were precleared with protein A/G agarose beads (Santa Cruz Biotechnology, Santa Cruz, CA, USA) for 1 h at 4°C. The precleared lysates were rotation mixed overnight at 4°C with 2 μg of rabbit anti-TET1 (Santa Cruz) or rabbit anti-PARP-1 (Santa Cruz, H-250) primary antibodies. Samples were further incubated with protein A/G agarose beads for 4 h at 4°C. The beads were pelleted by centrifugation, washed and immunoprecipitated proteins were eluted by boiling beads in sample buffer for 5 min. Proteins were separated by tris-glycine SDS-PAGE electrophoresis on 12% PAA gel, transferred to a PVDF membrane and analysed by immunoblotting using rabbit anti-TET1 (Santa Cruz) or rabbit anti-PARP-1 (Santa Cruz, H-250) primary antibody and mouse anti-rabbit IgG (Conformation Specific) (Cell Signaling, L27A9) HRP Conjugate secondary antibody.

**Slot-blot**

Genomic DNA was extracted from NIH3T3 cells by Bio-On-Magnetic-Beads (BOMB) protocol[1]. The concentration of DNA was determined by NanoPhotometer n60 (Implen) and each sample was diluted to the concentration of 10 ng/μl and denatured in 0.4 M NaOH, 10 mM EDTA at 95°C for 10 min. The denatured samples were subsequently neutralized with ice-cold 2 M ammonium acetate (pH 7.0) and 400ng of each DNA sample was applied on a Hybond N+ positively charged nylon membrane in an assembled Bio-Dot apparatus (Bio-Rad) according to the manufacturer’s instructions. The same amount of DNA that was not denatured was also applied on the membrane for evaluation of total DNA by methylene blue staining (as the staining is more robust with non-denatured DNA). The membrane was baked at 80°C for 30 min and cut in half so that the first half contained all of the samples as denatured DNA and the second half contained the same non-denatured samples. The first half of the membrane was blocked by 5% milk for 1 h at room temperature and incubated with rabbit anti-5hmC (1:10000, Active Motif) overnight at 4°C. This was followed by 1h incubation with horseradish peroxidase-conjugated secondary anti-rabbit antibody (Abcam, 1:20000) at room temperature. The second half of the membrane (with non-denatured DNA samples) was stained by 0.1% methylene blue (in 0.5 M sodium acetate pH 5.2). Chemiluminescence and methylene blue staining were captured by iBright FL1500 Imaging System. Band intensities were analysed with ImageJ 1.52p software using the _BandPeakQuantification plugin[2]. Obtained 5hmC background-corrected integrated intensities were normalised to the appropriate background-corrected integrated intensities of methylene blue stained total DNA for each sample.

**References**

1. Oberacker P, Stepper P, Bond DM, Höhn S, Focken J, Meyer V, et al. Bio-On-Magnetic-Beads (BOMB): Open platform for high-throughput nucleic acid extraction and manipulation. PLoS Biol. 2019;17:e3000107. doi:10.1371/journal.pbio.3000107.

2. Ohgane K. Quantification of Gel Bands by an Image J Macro, Band/Peak Quantification Tool. ProtocolsIo. 2019;:1–12. doi:dx.doi.org/10.17504/protocols.io.7vghn3w.
